# *MM-MDS*: A Multidimensional Scaling Database with Similarity Ratings for 240 Object Categories from the *Massive Memory* Picture Database

**DOI:** 10.1371/journal.pone.0112644

**Published:** 2014-11-12

**Authors:** Michael C. Hout, Stephen D. Goldinger, Kyle J. Brady

**Affiliations:** 1 Department of Psychology, New Mexico State University, Las Cruces, New Mexico, United States of America; 2 Department of Psychology, Arizona State University, Tempe, Arizona, United States of America; 3 Department of Human Systems Engineering, Arizona State University, Polytechnic, Mesa, Arizona, United States of America; University of Leuven, Belgium

## Abstract

Cognitive theories in visual attention and perception, categorization, and memory often critically rely on concepts of similarity among objects, and empirically require measures of “sameness” among their stimuli. For instance, a researcher may require similarity estimates among multiple exemplars of a target category in visual search, or targets and lures in recognition memory. Quantifying similarity, however, is challenging when everyday items are the desired stimulus set, particularly when researchers require several different pictures from the same category. In this article, we document a new multidimensional scaling database with similarity ratings for 240 categories, each containing color photographs of 16–17 exemplar objects. We collected similarity ratings using the *spatial arrangement method*. Reports include: the multidimensional scaling solutions for each category, up to five dimensions, stress and fit measures, coordinate locations for each stimulus, and two new classifications. For each picture, we categorized the item's prototypicality, indexed by its proximity to other items in the space. We also classified pairs of images along a continuum of similarity, by assessing the overall arrangement of each MDS space. These similarity ratings will be useful to any researcher that wishes to control the similarity of experimental stimuli according to an objective quantification of “sameness.”

## Introduction

Researchers across domains of cognitive science (and related fields) often require stimuli with varying degrees of similarity to one another. For instance, someone interested in visual search may wish to know whether attention is drawn to foils that are more or less similar to a designated target object [1]. Or a recognition memory investigator may wish to control the difficulty of a discriminating target and lure objects [2]. Empirically, however, manipulating or measuring the similarity of stimulus items can be a challenging task.

One approach is to employ simplistic stimuli, and vary a single feature of each item, such as the color or orientation of a rectangular bar [3]. This is a suboptimal approach with higher-level vision, however, because real-world objects contain many features that may be ill-defined or inconsistent across exemplars of a category. Restricting stimulus complexity to simple, arbitrary objects (e.g., rotated, colored shapes) permits tight experimental control; visual similarity across items is easily assessed and manipulated. But natural perception rarely involves objects defined by minimal arbitrary features, such as “red, 45-degree rotation, rectangular.” To help researchers better address ecologically valid perception, we created a large-scale database, evaluating psychological similarity among multiple exemplars from 240 object categories. We used multidimensional scaling (MDS) to identify objects with varying degrees of similarity to one another (see [4] for a similar approach). One appealing aspect of MDS is that it allows people to provide similarity estimates based on whatever featural dimensions they consider salient or important. MDS does not require *a priori* identification of feature dimensions, or arbitrary rating schemes, such as ranking the similarity of colored bars based on degree of rotation, or distance in RGB color space.

We collected similarity ratings on pictures of real-world objects; things that people encounter in everyday life, defined by many features simultaneously. These stimuli afford researchers flexibility in item selection, as they can choose pictures with featural variation without compromising the categorical identity of the exemplars. All stimuli came from the “Massive Memory” database ([5], [6]; cvcl.mit.edu/MM/stimuli.html). We selected a large variety of semantically-matched pictures: 200 object categories were represented by 17 individual exemplars each, and another 40 categories by 16 exemplars each. We then obtained MDS solutions for each image category (e.g., an MDS space of 17 coffee mugs, or 16 lamps). These “psychological spaces” were used to identify stimulus pairings with varying degrees of similarity. The logic is simple: By using the MDS spaces, pairs of images can be identified that are similar or dissimilar, indexed by their proximity in psychological space. We were thus able to define pairwise similarity along a continuum of psychological distances. This technique is appealing because it provides similarity ratings that are psychologically grounded, rather than defined arbitrarily, and because the large sets of category exemplars provide many stimulus pairings for researchers to draw upon.

### Similarity ratings and multidimensional scaling

MDS is a tool by which researchers can obtain quantitative estimates of the similarity among groups of items [7]. More specifically, MDS is a set of statistical techniques that takes as input item-to-item similarity ratings. It then uses data-reduction procedures to minimize the complexity of the similarity matrix, permitting (in many cases) a visual appreciation of the underlying relational structures that were used to govern the similarity ratings.

Likely the most common method for obtaining similarity estimates is simply to ask people to numerically rate object pairs via Likert scales. In this technique, ratings are collected for every possible pairwise combination of stimuli (e.g., “respond ‘1’ when the items are most similar and ‘9’ when the pair is most dissimilar”). This *pairwise method* is useful and simple to implement. However, when the set of stimuli to be scaled is large, this technique is not ideal: The number of comparisons necessary to fill an item-to-item similarity (or *proximity*) matrix grows rapidly as a function of stimulus set size, leading to lengthy experimental protocols. Data collection becomes cumbersome, and concerns therefore arise regarding the vigilance of the raters [8].

An alternative way to collect similarity estimates is the *spatial arrangement method* (*SpAM*), originally proposed by Goldstone ([9]; see also [10]). This technique is faster and more efficient than the pairwise method, and produces output data of equal quality, relative to its more well-established counterpart [8]. Here, many (or all) of the to-be-rated stimuli are presented at once, and participants move the items around on the computer screen, placing them at distances from one another that reflect subjective similarity estimates (items that are rated as similar are placed close to one another, and dissimilar items are placed proportionately farther away). The task can be conceptualized as having people project their own psychological spaces onto a two-dimensional plane (i.e., the computer screen). Once the participant has finished organizing the space, a proximity matrix is derived from the pairwise Euclidean distances (measured in pixels) between every pair of items. This technique is extremely well-suited for collecting large quantities of MDS data in relatively short periods of time (a set of 17 stimuli would be scaled in roughly 20 minutes using the pairwise method, whereas SpAM would likely be completed in under 5 minutes).

## Methods

### Ethics

This research was approved by the Institutional Review Board of Arizona State University (on 2/9/2009, IRB Protocol #0901003647), and was considered “exempt” in accordance with Federal regulations, 45 CFR Part 46. Informed consent was obtained, prior to the start of the experiment, by providing research participants with a written cover letter, detailing the aim and scope of the research, and informing them that their data could not be linked to them directly, or through indirect identifiers. The cover letter stated that a spacebar keypress (on the experimental computer) would be taken as their consent to participate, and that they could choose to withdraw from the study at any time, without penalty. Written consent was not obtained because the IRB waived the requirement for the investigators to obtain a signed consent form, under article 46.117, section C (of the Code of Federal Regulations, Protection of Human Subjects), finding that this research “present[ed] no more than minimal risk of harm to subjects and involve[d] no procedures for which written consent is normally required outside of the research context.” Thus, initiating of the experiment served to document the participant's consent to participate. This entire procedure was approved by the IRB at Arizona State University.

### Participants

Two-hundred and forty students from Arizona State University participated as partial fulfillment of a course requirement. Additionally, ten undergraduate research assistants (from the *Memory and Language Laboratory* at Arizona State University) participated voluntarily. All participants had normal or corrected-to-normal vision, and all reported normal color vision.

### Design

One-hundred and fifty participants completed an experiment wherein they were given 15 SpAM trials. On each trial, a new category of images was shown to the participant. Selection of image categories was counterbalanced, such that for every 16 participants, all 240 categories were scaled exactly once. We also collected a smaller amount of data from 90 participants. These people completed the same task, but were only administered 10 SpAM trials (with randomly selected categories). This shortened version of the task was given because the SpAM task was appended to an unrelated experiment. Finally, the 10 research assistants that participated in the experiment completed 16 sessions (with 15 SpAM trials per session), over the course of an entire semester. Thus, each of these participants scaled every one of the 240 image categories exactly once. For each image category, between 15 and 24 participants contributed similarity ratings (*M* = 19.48).

### Stimuli

The stimuli were photographs of real-world objects, resized (maintaining original proportions) to a maximum of 2.5° of visual angle (horizontal or vertical), from a viewing distance of 55 cm. Images were no smaller than 2.0° of visual angle along either dimension. The pictures contained no background; a single object or entity was present in each image (e.g., an ice cream cone, a pair of shoes). A full list of stimulus categories (along with complete summary statistics for each) can be found in the sorting table available in the online database.

### Apparatus

Data were collected on up to 12 computers simultaneously, all with identical hardware and software profiles. Dividing walls separated each subject station, and experimental sessions were monitored at all times by one or more research assistants. The PCs were Dell Optiplex 380 systems (3.06 GHz, 3.21 GB RAM) operating at 1366×768 resolution on Dell E1912H 18.5′′ monitors (operated at a 60 Hz refresh rate). Displays were controlled by an Intel G41 Express chipset, and the operating systems were Windows XP. E-Prime v2.0 Professsional software [11] was used to control stimulus presentation and collect responses.

### Procedure

On each trial, a new category of images was shown to the participant, arranged in discrete rows, with random item placement. People were instructed to drag and drop the images in order to organize the space such that the distance among items was proportional to each pair's similarity (with closer in space denoting greater similarity). Participants were given as much time as they needed to scale each category; typically, trials lasted between 2 and 5 minutes. Once participants finished arranging the items, a right mouse-click completed the trial. In order to avoid accidental termination of the trial, participants were then asked if they were satisfied with the space, or if they needed more time (responses were indicated via the keyboard).

## Results

### MDS algorithm

For each data set, we used the *INDSCAL* scaling algorithm, which is a version of the *ALSCAL* algorithm that also provides individual differences metrics (see [12], [13]), via SPSS 22.0 [14]). This algorithm uses an alternating least-squares, weighted Euclidean distance model, and can accommodate multiple data sources (i.e., multiple participants). Our data were analyzed using metric MDS: The dissimilarity matrices were treated as ratio-level data, because the scores were distances between points, measured in pixels. The stress measurement for this algorithm is computed using Kruskal's stress formula 1. The iterative scaling procedure concludes when one of three criteria have been met: 1) Stress fails to decrease by more than.001 across iterations; 2) stress falls below.005; or 3) a maximum of 30 iterations have been completed.

### Dimensionality of the MDS space

In order to determine the appropriate dimensionality in which the data should be scaled, analysts often rely on the *stress* of the solutions. A common approach is to create *scree plots*, which display stress as a function of dimensionality. Stress typically decreases with the addition of each dimension, but a useful heuristic is to look for the “elbow” in the plot; the value at which added dimensions cease to substantially improve model fit [15], [16]. In the event that an elbow is not clearly pronounced, researchers may rely on prior hypotheses regarding the likely dimensionality of the space, and must also consider the trade-off between a high-dimensional space, and the ability (and importance) to visualize the results. When visualization is unimportant, high-dimensional spaces can be useful, but when the analysis is purely exploratory, two- or three-dimensional spaces may be preferred, because they allow straightforward visual inspection of the data. Typically, dimension selection is performed conservatively, because increasing the dimensionality of the MDS solution is not universally beneficial. Choosing the correct dimensionality, therefore, often depends on stress levels and on interpretability [17]. In essence, one strives to strike a balance between finding a “good” solution, and one that is interpretable. Finally, recent empirical approaches to dimension selection have been developed that use Bayesian mathematics to address the trade-off between the complexity of the space and the fit of the solution, relative to the original similarity estimates (see [16], [18]).

For each of the data sets, we provide the full results of scaling in dimensions 1–5, including the coordinate points, stress measures, and R^2^ values (which provide a measure of the proportion of variance accounted for by the solution). Stress and explained variance are also graphically plotted, to allow for visual inspection of these metrics as a function of dimensionality.

### Sample analysis

**Figure 1:**
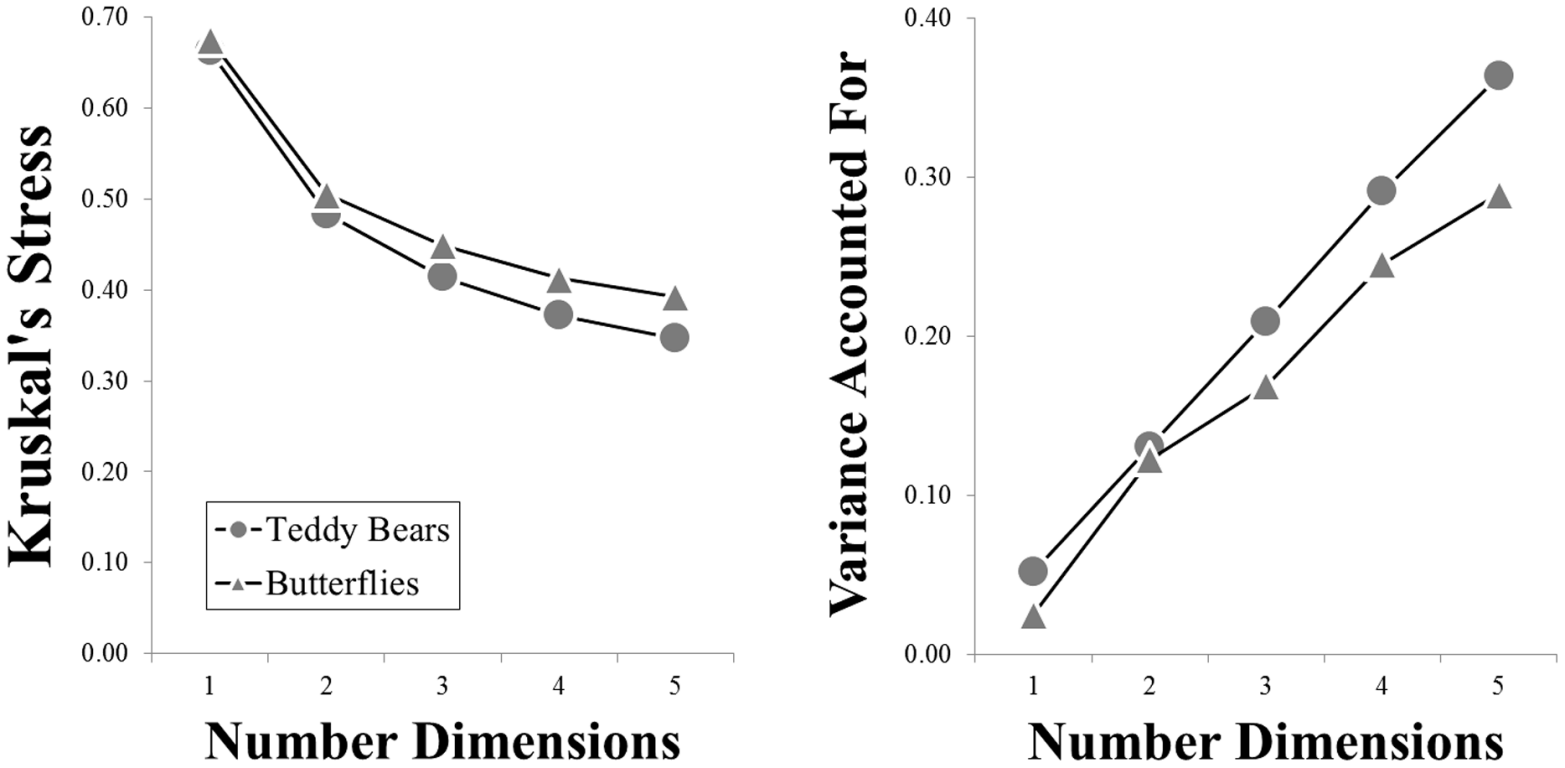
Stress (left) and proportion of variance accounted for (right), plotted as a function of the dimensionality of the space, for the teddy bear (circular symbols) and butterfly (triangular symbols) categories.

The quantity of the MDS data prevents a full report of the results here. The complete analyses can be found on the first-author's website (www.michaelhout.com), available for free download. Below, we provide a sample analysis on two of the stimulus sets (teddy bears and butterflies), for demonstration purposes. All data were analyzed in identical fashion. Nineteen volunteers contributed data for the teddy bear category, and 20 for the butterflies. Figure 1 shows stress and the proportion of variance accounted for (plotted as a function of dimensionality of the solution) for both stimulus sets. Clearly, stress is reduced to the largest degree moving from a single dimension to two dimensions, but the addition of third (and perhaps fourth) dimensions also appears to be meaningful, for both data sets. As is common, the stress plots show a sharp initial decrease, followed by a plateauing of model fit at higher dimensions, and by contrast, the proportion of explained variance in the space increases linearly with added dimensions.

**Figure 2:**
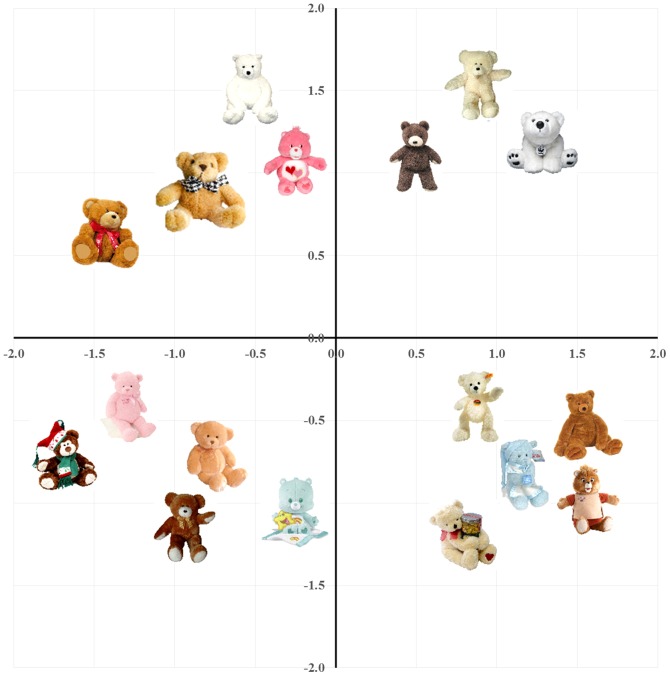
Two-dimensional MDS solutions for the teddy bear stimuli.

**Figure 3:**
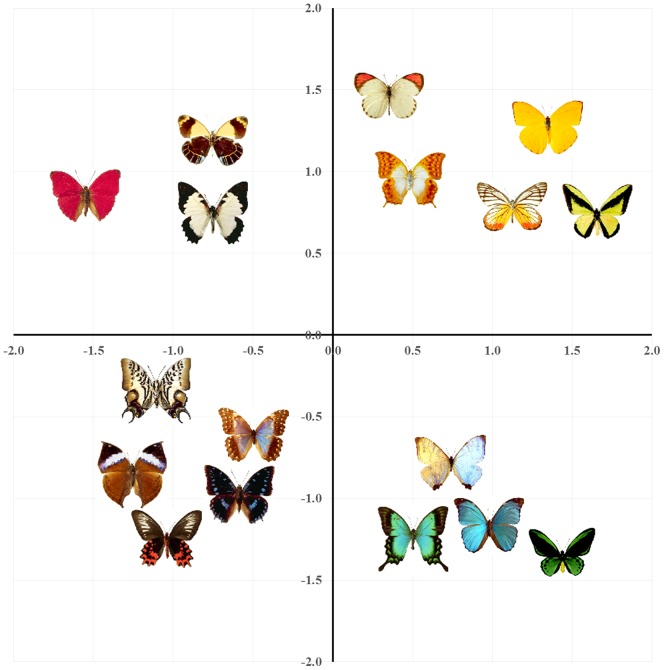
Two-dimensional MDS solutions for the butterfly stimuli.

Figures 2 and 3 show sample two-dimensional MDS plots of the stimuli. Dimension 1 is the primary dimension (i.e., it explains the most variance), followed in order by Dimensions 2, 3, 4 and 5 (for higher-dimensional solutions). These two-dimensional plots allow easy visual inspection of the spaces, but the scree plots suggest that the correct dimensionality of both spaces is likely to be three- or four-dimensions, which, when added to the solution, would add more information regarding relations among the pictures.

### Individual differences: “Weirdness” scores

As in any investigation of psychological similarity, the present participants were likely to exhibit individual differences in their appreciation of various stimulus characteristics, and the salience (or weight) assigned to those characteristics. For instance, given the teddy bear stimuli (Figure 2), participants may have appreciated the colors of the bears (dark browns and black vs bright blues and pinks), their postures (sitting vs. standing), their pattern uniformity (simple, plain-colored bears vs. more decorative ones), and so on. It is unlikely that every participant would use these features in identical fashion. Some participants may feel that color similarity is a dominant feature (as clearly shows up in the aggregate data for the butterflies, shown in Figure 3), whereas others may group items with respect to pattern uniformity. Indeed, some participants may have appreciated dimensions that others ignored entirely (e.g., whether a bear was wearing clothing). Although individual differences can sometimes be problematic, they are often helpful in investigating similarity using MDS. When any given participant performs the experiment, s/he likely focuses on a few features that drive an intuitive sense of similarity among stimuli. When these diverse ratings are aggregated across participants, MDS can reveal higher dimensional relationships, all of which are accordingly weighted by their importance to the group as a whole (see [8], for a more complete discussion of individual differences in MDS, as well as a newly proposed method for identifying outlier participants).

In order to provide a simple measure of individual differences in this large data set, we implemented the INDSCAL procedure within the ALSCAL scaling framework. This option reports “weirdness” scores for each raw similarity matrix (i.e., each participant). These scores take into account the relative importance of each dimension for each participant, and can range from 0 to 1. A participant whose similarities weight each dimension equivalently to the group average weights is assigned a weirdness value of zero. To the degree that any participant deviates from the average weighting, their weirdness value will approach one (e.g., a participant with one large weight and many low weights would receive a high weirdness score). In effect, these scores index individual differences by indicating the similarity of an individual's dimension weights relative to the group representation. We report these scores for each matrix for the scaling attempts in dimensions 2–5; because these scores rely on relative dimensional weighting, they cannot be calculated for one-dimensional solutions.

**Figure 4:**
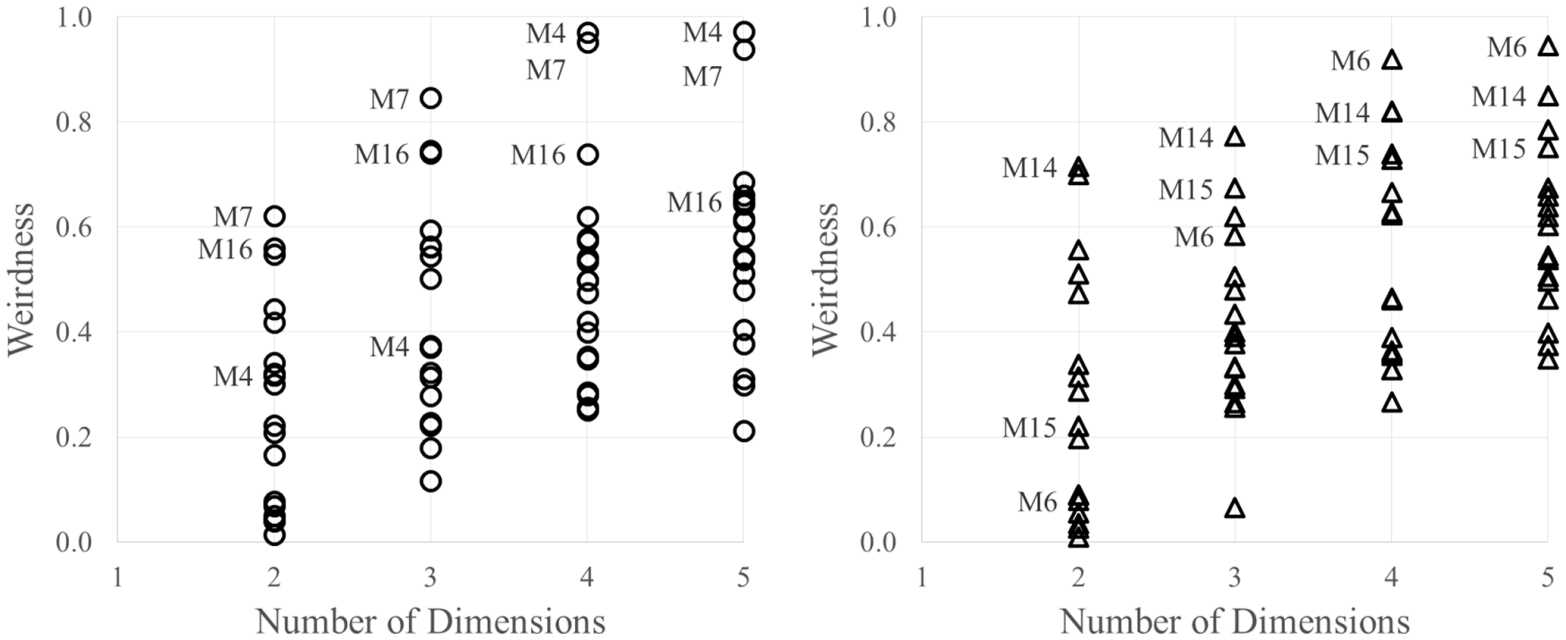
Weirdness scores for the teddy bear (left) and butterfly (right) categories, plotted as a function of dimensionality of the space (weirdness scores are not possible for one-dimensional solutions). Each symbols shows the score for one participant (i.e., one data matrix), and the three participants with the highest mean weirdness scores have been identified with labels, for demonstrative purposes.

Figure 4 displays the weirdness scores for the teddy bears and butterflies, as a function of dimensionality. Each symbol represents the score for one participant per solution, and the three participants with the highest mean weirdness scores (overall) are labeled. As can be seen for the teddy bears, Matrices 7 and 16 have weirdness scores that fall above the rest of the group at lower dimensionalities, whereas Matrices 4 and 7 are the most unlike the rest at the higher dimensionalities. By contrast, for the butterflies, Matrix 14 has high weirdness values at all dimensionalities, but the overall distribution of scores shows few clear outliers. The full report includes these scores for every category, so analysts can use whatever criteria they choose to identify potential outliers (e.g., participants with mean weirdness scores more than 2.5 standard deviations above the group mean). The full raw data matrices are also available for download, so if one wishes to exclude participants for any reason and rescale the results, this is readily possible.

### Identification of item pairings along a continuum of similarity

With the coordinates obtained from each psychological space, it is possible to identify object pairs that are more or less similar to one another, relative to all other possible pairs. No basic unit of measurement is present in MDS, so the inter-item distance values are expressed in arbitrary units. This makes it impossible to define numerical cutoff values for classifying pairs as “very similar” or “very dissimilar.” To provide empirically-driven identification of item pairs, tailored to each individual space, the followed procedure was employed: First, for each MDS space (in all five dimensionalities), a vector of distances was obtained, corresponding to the Euclidean distances in psychological space for all item pairs. For each 17-item category, there were 136 inter-item distances; for each 16-item category, there were 120 inter-item distances. Next, the distances were rank-ordered, and categorized as “close,” “mid,” or “far,” based on a ternary-split of all distances. For each pair of images, we provide the Euclidean distance in *k*-dimensional space, the ordinal ranking of the pair (where 1 is the most similar pair, with higher numbers indicating more distally placed pairs), and the classification of the pair (close, mid, far). The classification is provided as a convenience to researchers who wish to identify item pairs quickly, but with the full distance and ranking values, analysts can select item pairs however they so choose.

**Figure 5:**
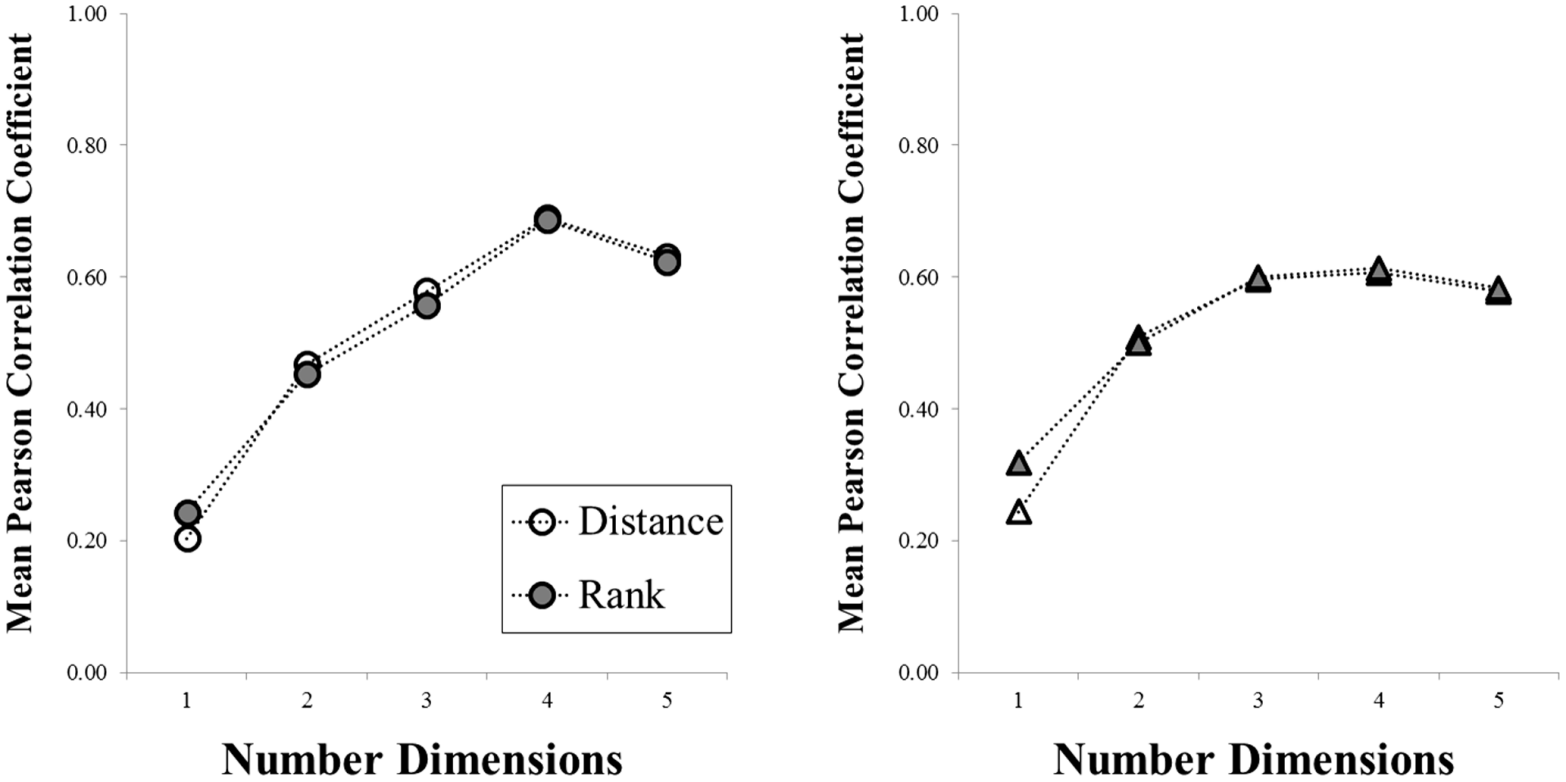
Organizational agreement curves for the teddy bear (left) and butterfly (right) categories, plotted separately for raw distances (open symbols) and ordinal rankings (closed symbols). Plotted are the mean Pearson correlation coefficients that relate the inter-item distance vectors across dimensionalities 1–5.

Another metric of interest is how consistent the stimulus organizations are across solutions with different dimensions. That is, if two particular objects are close together in a two-dimensional solution, will they remain close in a three- or four-dimensional solution? To estimate the degree to which rankings stayed consistent across solutions, we correlated the inter-item distance vectors across all solutions, then plotted “agreement curves” which show the average Pearson correlation coefficient for each dimensionality (e.g., the average correlation for a one-dimensional solution, relative to dimensions 2–5, and so on). In the full results, we provide (for each category) these correlations based on raw distances between items, and also the ordinal rank-ordering of pairs (and graphically display the results for easy visual inspection). Figure 5 shows the results for the teddy bears and butterflies. Note that the lower dimensionalities tend to have lower mean correlations, but the solutions become more stable at higher dimensions.

### Classification of item prototypicality (centrality)

Each image was also assigned a prototypicality (or centrality) rating. The idea is to identify items that are more or less central in the space, by examining how far they tend to be located relative to the other items. To calculate these classifications, we took the average distance from each item to every other item in the space. Items that fall in the center of the space tend to be close to others, so they receive a low average distance rating. Items that are proportionately farther out in the space tend to be further away from more of their partners, and so tend to receive a higher average distance rating. Once averages were calculated for each image, the items were rank ordered and classified based on a ternary split. The 1/3 of items with the smallest average distance were classified as “inner” items. The middle 1/3 of items were classified as “mid”, and the 1/3 of items with the highest average distances were classified as “outer” items. For each picture, we report the average distance, the ranking (with smaller numbers indicating the smaller average distances), and the classification (for each of the 5 dimensionalities).

**Figure 6:**
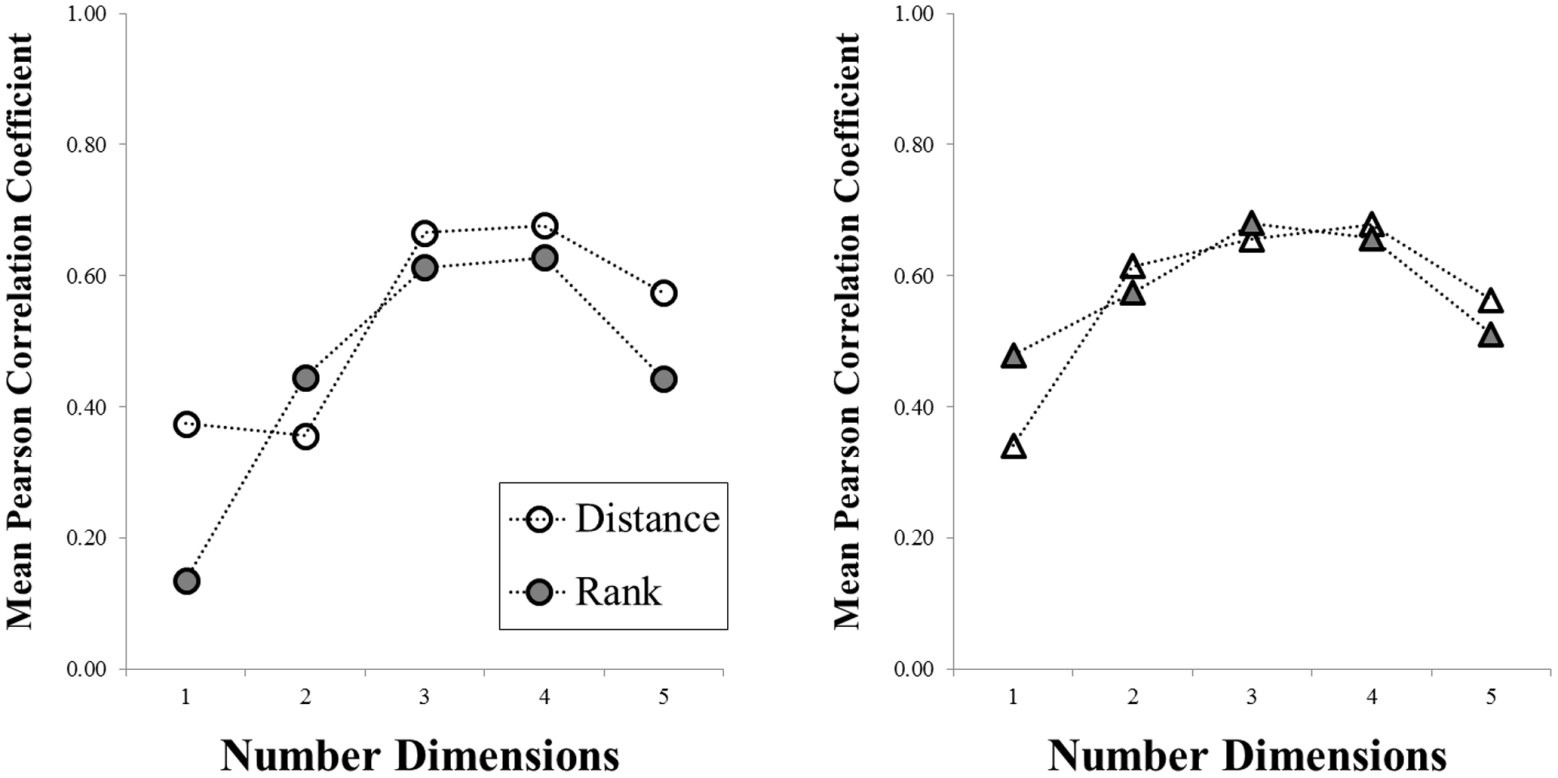
Prototypical agreement curves for the teddy bear (left) and butterfly (right) categories, plotted separately for raw distances (open symbols) and ordinal rankings (closed symbols). Plotted are the mean Pearson correlation coefficients that relate the prototypicality ratings across dimensionalities 1-5.

As with the organizational stability of the spaces, it may be useful to know the consistency of prototypicality ratings across dimensionalities. The question now becomes, “how likely is an item that is centrally located in low-dimensional space to also be centrally located in high-dimensional spaces?” Once more, we created agreement curves, which now show the average Pearson correlation coefficients for item rankings (using raw distances and ordinal rankings) across dimensionalities (the full results graphically display these values). Figure 6 shows the results for the teddy bears and butterflies.

### Monte Carlo Simulations

In any investigation of similarity, it can be difficult to quantify the “quality” of any participant's solution, because similarity is often inherently subjective. Stress (and variance accounted for) provides an objective measurement, showing how well the scaling algorithm was able to accommodate the raw similarity scores across participants, but there is never any “right” answer regarding humans' intuitive sense of similarity. This creates an interesting dilemma: Even if there is little agreement across participants regarding similarity relations among some set of objects, an MDS algorithm can sometimes derive a group-level solution with low stress. Thus, it can be difficult to know whether any given solution faithfully reflects people's shared sense of inter-object similarity, rather than random noise. To address this concern, we performed Monte Carlo simulations, wherein random similarity data were generated, creating numerous matrices that shared no underlying structure (unlike, we presume, human-generated similarity estimates). We then compared the MDS results for these simulated data matrices to a subset of our real data.

### Method of the Monte Carlo simulations

We generated random data using two methods. The first was designed to mimic the SpAM procedure (which our real participants performed). For each simulated subject, a random configuration of 17 points was generated, located on a hypothetical two-dimensional plane, proportional to the monitor size used by real participants. The item placements were then used to derive data matrices containing the two-dimensional Euclidean distances between all item pairs. The second method was designed to mimic the standard, pairwise method of collecting similarity estimates, wherein participants see two items at a time, and provide a similarity estimate for each pair (usually using a Likert scale). Here, for each simulated participant, we randomly generated similarity ratings for each item pair, one pair at a time. For both methods, there were 17 items per space, giving rise to 136 total proximities.

The reason for including the pairwise simulation method was to provide an additional point of comparison to our real data. Importantly, we expected the results of the pairwise method to underperform the first. When using SpAM, all stimulus items are shown at once, and thus the context remains entirely consistent: Participants are compelled to consider the entire stimulus set when moving items around on the screen. For instance, if a person feels that item *A* is similar to item *B* (indicated by placing them close together), and that item *B* is similar to item *C*, then because of the two-dimensional plane used to convey ratings, she will also indicate that item *A* is relatively similar to item *C*. If this arrangement of points disagrees with her sense of similarity for these three items (or others in the set), she must reconsider her placement of items in space. Simply put, the ratings provided for any two stimuli must also take into account the similarity of those items relative to all others in the set.

By contrast, with the pairwise method, the overall stimulus context is challenging to appreciate, because different items are shown over and over again in (relative) isolation. Thus, a person might violate the integrity of her overall solution by indicating that items *A* and *B* are similar on trial *n*, that items *B* and *C* are similar on trial *n+1*, and that items *A* and *C* are *dis*similar on trial *n+2*. This creates a problem (i.e., stress) for the MDS algorithm to accommodate, because the individual rankings may contradict each other in context of the entire stimulus set. We expected this additional problematic variation in the simulated scores to decrease the quality of the scaled solutions, despite those scores being generated from a random process.

Therefore, conceptually, the first set of simulations model situations in which participants, without thinking, randomly placed the 17 items in various locations on the screen. By contrast, the second set of simulations model participants who may have given arbitrary numerical ratings to pairs of stimuli, one pair at a time. By including both sets of simulations, we can examine the extent to which our participants' data outperform the truly random data (i.e., the pairwise simulations), and the degree to which the constraints of the data collection method (i.e., the spatial arrangement method) confer an advantage by protecting against such integrity violations. To preview our results, we found that the spatial arrangement method does afford an advantage (indexed by, for instance, lower stress values and higher variance accounted for), but that our real participants' data are markedly improved, relative to both types of random simulated ratings. This indicates that the empirical data are systematic, and reflect meaningful similarity ratings, rather than random noise. For thoroughness and consistency with the rest of the database, we report the results of the Monte Carlo simulation weirdness scores and agreement curves (for inter-item distance vectors and prototypicality), but the important findings are those pertaining to stress and variance accounted for.

### Results of the Monte Carlo simulations

For each Monte Carlo method, we performed 20 simulations, each with 20 simulated participants and 17 objects. These simulations were compared to our real data from categories 41 through 60 (which were the first 20 categories to have 17 exemplars). These matrices are provided for download, alongside the real participants' data. We analyzed stress, variance accounted for, mean weirdness scores, and the agreement curves (for inter-item distance vectors and prototypicality). All data, except weirdness scores, were analyzed using 3 (Data Type: real, SpAM simulation, pairwise simulation) X 5 (Dimensionality: 1–5) mixed-model, repeated- measures ANOVAs. Data Type was the only between-subjects factor. For weirdness scores, the design was 3 X 4, because they cannot be provided for one-dimensional solutions.

#### Stress

**Figure 7:**
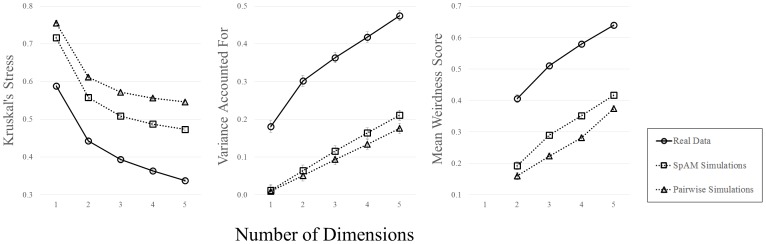
Stress (left), variance accounted for (center), and mean weirdness scores (right), plotted as a function of dimensionality. Data are plotted separately for real data (circular symbols, solid lines), SpAM simulations (square symbols, dotted lines), and pairwise simulations (triangular symbols, dotted lines). Error bars represent ±1 standard error of the mean.

We found main effects of Data Type, *F*(2, 57) = 360.85, *p*<.001, *n^2^_p_* = .93, and Dimensionality, *F*(4, 54) = 1379.43, *p*<.001, *n^2^_p_* = .99. As predicted, stress values were the lowest for the real data (.43), followed by the SpAM (.55) and pairwise simulations (.61). Planned, Bonferroni-corrected comparisons revealed that each group was significantly different from the others (all *p*<.001). As is typical, stress values decreased with added dimensions (.69,.54,.49,.47, and.45 for dimensions 1–5, respectively). The interaction of factors was also significant, *F*(8, 108) = 18.98, *p*<.001, *n^2^_p_* = .58, indicating that the real data exhibited the sharpest decrease in stress with added dimensions (a reduction of.25 from dimensions 1–5), relative to SpAM (a reduction of.24), and pairwise simulations (a reduction of.21). The results are plotted in Figure 7.

#### Variance accounted for

We found main effects of Data Type, *F*(2, 57) = 109.27, *p*<.001, *n^2^_p_* = .79, and Dimensionality, *F*(4, 54) = 699.19, *p*<.001, *n^2^_p_* = .98. As we expected, the most variance was accounted for in our real data (.35), followed by SpAM (.11) and pairwise simulations (.09). Planned comparisons revealed differences between the real data and both simulations (*p*s<.001), but that simulations were not different from one another. Variance accounted for increased with added dimensions (.07,.14,.19,.24, and.29 for dimensions 1–5, respectively). The interaction was significant, *F*(8, 108) = 16.14, *p*<.001, *n^2^_p_* = .54, indicating a sharp increase in variance accounted for in the real data (an increase of.29 from dimensions 1–5), and shallower increases for the SpAM (.20) and pairwise (.17) simulations (see Figure 7).

#### Mean weirdness scores

There were main effects of Data Type, *F*(2, 57) = 310.73, *p*<.001, *n^2^_p_* = .92, and Dimensionality, *F*(3, 55) = 568.25, *p*<.001, *n^2^_p_* = .97. The highest weirdness scores were obtained for the real data (.53), followed by SpAM (.31) and pairwise (.26) simulations, and planned comparisons revealed that all groups were different from one another (*p*s<.001). Weirdness increased with added dimensions (.25,.34,.40, and.48 for dimensions 2–4, respectively), and the interaction of factors was significant, *F*(6, 110) = 3.46, *p*<.01, *n^2^_p_* = .16. This indicates that the real data had the sharpest increase in weirdness scores (an increase of.23 from dimensions 2–4), followed by SpAM (.22) and pairwise (.21) simulations (see Figure 7).

This seemingly paradoxical effect (i.e., that weirdness was higher for real data) arises because the simulated data has no real underlying structure, and thus all simulated participants more closely resemble one another, relative to real participants who are prone to individual differences in their overall sense of similarity (and thus their relative weighting of the dimensions). With meaningful empirical data, individual differences are arise with respect to the degree to which the aggregate solution reflects the ratings of any one participant, particularly when higher dimensionalities are considered (because with additional dimensions come additional opportunities for disagreement). Random data is unlikely to exhibit differential weighting of the dimensions across simulated participants (i.e., higher weirdness scores) because the dimensions themselves do not represent meaningful variation in the similarity scores. As such, the importance of each dimension will vary when examining real data, but is likely to be uniform when the data are randomly produced.

#### Inter-item distance vectors

**Figure 8:**
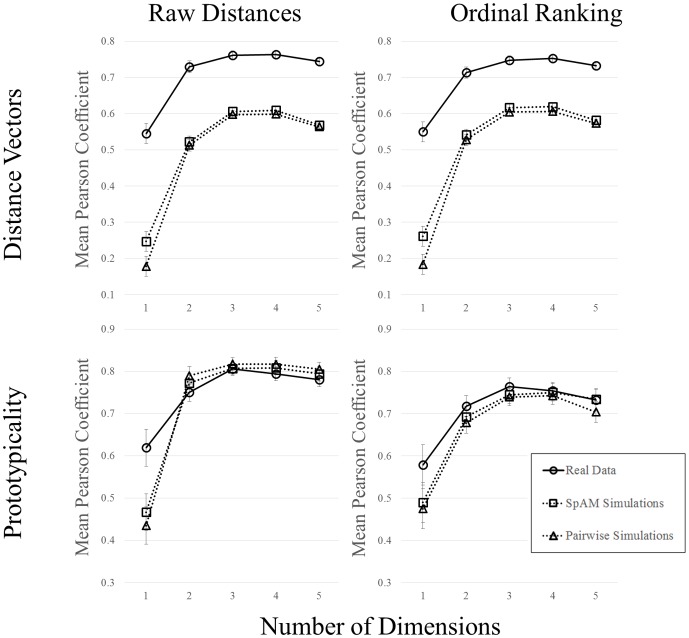
Agreement curves for inter-item distance vectors (top), and prototypicality (bottom), plotted as a function of dimensionality. Data are shown separately for the raw distance values (left) and ordinal rankings (right). Separate lines are shown for real data (circular symbols, solid lines), SpAM simulations (square symbols, dotted lines), and pairwise simulations (triangular symbols, dotted lines). Error bars represent ±1 standard error of the mean.

For the raw distance values, we found main effects of Data Type, *F*(2, 57) = 71.97, *p*<.001, *n^2^_p_* = .72, and Dimensionality, *F*(4, 54) = 294.24, *p*<.001, *n^2^_p_* = .96. The highest agreement was obtained for the real data (.71), followed by the SpAM (.51) and pairwise (.49) simulations. Planned comparisons revealed that the real data were improved relative to both simulations (*p*s<.001), but that the simulations were not different from one another. Agreement curves showed a bow, with higher agreement at the middle dimensions (.32,.59,.66,.66, and.63 for dimensions 1–5, respectively). This is not surprising, considering that the organization of the upper and lower dimensionalities are on the extremes, and are therefore being correlated with solutions that are likely to differ most substantially, relative to their own. The interaction of factors was also significant, *F*(8, 108) = 8.65, *p*<.001, *n^2^_p_* = .39. We also performed the same analysis on the ordinal rankings of item pairs. However, the raw distance values are a more sensitive measure, and the pattern of results for ordinal rankings was identical, so we do not report them here. See Figure 8 for plots of both indices.

#### Prototypicality rankings

For the raw distances, we found no main effect of Data Type, *F*(2, 57) = .27, *p* = .77. This somewhat surprising effect is easily explained. Because the simulated data have no real underlying structure, adding dimensions to the space does not actually extract any new, meaningful information about the organization of the space. This makes it likely for an object to maintain its relative position in the space across dimensionalities (and by implication, makes it likely that the agreement curves will indicate high coherence). There was a main effect of Dimensionality, *F*(4, 54) = 71.24, *p*<.001, *n^2^_p_* = .84, indicating a similar bow in the agreement curves at the middle dimensions (.51,.77,.81,.81, and.79 for dimensions 1–5, respectively). The interaction was also significant, *F*(8, 108) = 3.40, *p*<.01, *n^2^_p_* = .20. We performed the same analysis for the ordinal rankings and found the same pattern of results for the main effects, but a non-significant interaction of factors (see Figure 8).

## Discussion

To create the present database, we collected a large amount of similarity ratings on a variety of real-world item categories [5], [6]. Our goal was to acquire a set of psychological spaces that could be used by experimental psychologists to select item pairs across a range of visual similarity. This information can be used by attention and perception researchers, those interested in categorization, or those wishing to control the similarity of materials for any purpose. It bears mentioning that there is, of course, no single “correct” notion of similarity, and that researchers in different domains may use different criteria to choose stimulus categories for their work. For instance, researchers interested in primarily visual similarity may wish to use stimulus categories that vary primarily in visual features (e.g., the butterflies, which share the same general shape but differ in color). By contrast, researchers interested in more conceptual features may choose categories that vary in semantic features (e.g., the teddy bears, which vary in color, but also posture, clothing, and whether they are holding something). We hope that the database will be useful to a wide range of researchers.

For each stimulus set, we provide stress and explained variance measures for solutions in dimensions 1–5, and the coordinate locations for each item across dimensions. We also provide weirdness scores, which estimate the individual differences in our data. Finally, we classified each pair of items along a continuum of psychological similarity, classified each item according to its centrality (prototypicality) in its MDS space, and we calculated agreement curves showing how these constructs change across solutions plotted in various dimensionalities.

In order to provide an objective baseline to evaluate our data, we conducted Monte Carlo simulations in which we generated random data in manners analogous to the SpAM and pairwise methods (see [8]). We found that our real data exhibited lower stress values, more explained variance, and greater internal consistency, relative to either type of random data, suggesting that other researchers can use similar Monte Carlo simulation methods to evaluate how well group-level MDS data truly reflect shared notions of similarity across observers.

The full set of stimuli, analyses, and raw data can be found at the first-author's website: www.michaelhout.com, available for free download. We also included a large electronic table (in Microsoft Excel) that provides complete summary statistics for all categories. Researchers can use this table to sort the categories and find stimuli that suit their needs, based on whatever criteria they choose (e.g., stress levels, explained variance, individual differences metrics). Additionally, the software used to collect the similarity ratings is also freely available (written in both E-Prime and JAVA), as well as software for collecting similarity ratings in a variety of other manners. All data and software will be permanently hosted at that location.
